# Supplementary material for: The Perceived Impact of COVID-19 on Comfort Food Consumption over Time: The Mediational Role of Emotional Distress
Source: Nutrients. 2021 Jun 2;13(6):1910. doi: 10.3390/nu13061910 (PMC8228314; doi:10.3390/nu13061910)
Supplement: Supplementary file 1 [file nutrients-13-01910-s001.zip › nutrients-1214813-supplementary.pdf]

Table S1. Distribution of the study participants across waves.

**Variable**	**W1**<br>***n*= 1038**	**W2**<br>***n*= 509**	**W3**<br>***n*= 412**	**W4**<br>***n*= 475**	**W5**<br>***n*= 430**	**W6**<br>***n*= 415**
Age (SD)	29.52 (11.6)	30.03 (11.9)	30.97 (12.5)	30.86 (12.3)	31.11 (12.8)	30.71 (12.2)
Gender (Women)	69.0%	70.4%	69.3%	68.9%	69.5%	69.9%
Occupation (Students)	66.3%	65.2%	61.7%	62.4%	61.3%	61.6%

Table S2. Direct, indirect effects, confidence intervals, standard errors and mediation effects: Models 5 to 10.

	Direct effect	Indirect effects				
	COVID-19 Perceived impact (W1) → Comfort food	COVID-19 Perceived impact (W1) → Emotional distress → Comfort food	CI 95%	Standard error	Mediation %	Mediation type
Model 5	0.168	0.113	[0.021, 0.204]	0.047	40	Full
Model 6	0.355**	0.107	[0.018, 0.196]	0.045	23	Partial
Model 7	0.245*	0.113	[0.020, 0.206]	0.047	32	Partial
Model 8	0.451**	0.058	[-0.023, 0.140]	0.042	12	None
Model 9	0.306**	0.058	[-0.035, 0.151]	0.048	16	None
Model 10	0.297**	0.074	[-0.023, 0.170]	0.049	20	None

Note. * $p < .05$, ** $p < .01$.

Model 5 included Emotional distress on W3 and Comfort food on W4, $\chi2$ (41) = 88.637, p <0.001, CFI = 0.970, TLI = 0.960, RMSEA = 0.033 [90% CI = 0.024, 0.043].

Model 6 included Emotional distress on W3 and Comfort food on W5, $\chi2$ (41) = 71.678, p <0.001, CFI = 0.980, TLI = 0.973, RMSEA = 0.027 [90% CI = 0.016, 0.037].

Model 7 included Emotional distress on W3 and Comfort food on W6, $\chi2$ (41) = 107.684, p <0.001, CFI = 0.959, TLI = 0.945, RMSEA = 0.040 [90% CI = 0.031, 0.049].

Model 8 included Emotional distress on W4 and Comfort food on W5, $\chi2$ (41) = 60.503, p= 0.025, CFI = 0.988, TLI = 0.984, RMSEA = 0.021 [90% CI = 0.008, 0.032].

Model 9 included Emotional distress on W4 and Comfort food on W6, $\chi2$ (41) = 102.095, p <0.001, CFI = 0.965, TLI = 0.953, RMSEA = 0.038 [90% CI = 0.029, 0.047].

Model 10 included Emotional distress on W5 and Comfort food on W6, $\chi2$ (41) = 91.948, p <0.001, CFI = 0.970, TLI = 0.960, RMSEA = 0.035 [90% CI = 0.025, 0.044].
